# Supplementary material for: Effects of Near-Infrared Light on Well-Being and Health in Human Subjects with Mild Sleep-Related Complaints: A Double-Blind, Randomized, Placebo-Controlled Study
Source: Biology (Basel). 2022 Dec 29;12(1):60. doi: 10.3390/biology12010060 (PMC9855677; doi:10.3390/biology12010060)

Table S1 Demographics summer and winter. Demographics of the individuals in the different groups for summer and winter separately. Except for the number of males and females, all values are shown as average (SEM). Abbreviations used: h = hours, kg = kilogram SEM = Standard error of mean, ns = not significant, PSQI = Pittsburgh sleep quality index, ESS = Epworth sleepiness scale, BDI = Beck's Depression Inventory, BMI = Body mass index.

	**Winter**				**Mean Winter (SEM)**	**Summer**				**Mean Summer (SEM)**	**Significance (season)**
	0 J.cm^{-2}	1 J.cm^{-2}	4 J.cm^{-2}	6.5 J.cm^{-2}		0 J.cm^{-2}	1 J.cm^{-2}	4 J.cm^{-2}	6.5 J.cm^{-2}		
Number (M:F)	7 (1:6)	7 (2:5)	8 (3:5)	8 (3:5)	30 (9:21)	6 (3:3)	7 (3:4)	7 (4:3)	6 (3:3)	26 (13:13)	
Age (y)	39.6 *(5.8)*	42.0 *(5.6)*	41.8 *(4.7)*	40.4 *(5.3)*	40.9 *(2.5)*	36.0 *(3.5)*	34.4 *(3.3)*	35.1 *(5.8)*	33.3 *(3.9)*	34.7 *(2.0)*	ns
Chronotype (h)	3.9 *(0.6)*	4.2 *(0.5)*	3.7 *(0.4)*	3.8 *(0.4)*	3.9 *(0.2)*	4.2 *(0.4)*	4.3 *(0.4)*	5.1 *(0.4)*	4.8 *(0.5)*	4.6 *(0.2)*	< 0.05
Sleep duration (h)	7.3 *(0.8)*	7.5 *(0.6)*	7.6 *(0.5)*	6.5 *(0.4)*	7.2 *(0.3)*	6.7 *(0.4)*	7.2 *(0.3)*	7.2 *(0.3)*	7.3 *(0.3)*	7.1 *(0.2)*	ns
Sleep deficit (h)	0.8 *(0.3)*	1.3 *(0.3)*	0.7 *(0.2)*	1.0 *(0.3)*	0.9 *(0.1)*	2.1 *(0.7)*	2.0 *(0.5)*	1.4 *(0.3)*	2.2 *(1.1)*	1.9 *(0.3)*	< 0.01
PSQI	10.3 *(1.6)*	11.0 *(1.7)*	9.6 *(1.5)*	11.0 *(1.1)*	10.5 *(0.7)*	11.2 *(1.0)*	9.9 *(1.6)*	10.0 *(0.9)*	10.2 *(0.7)*	10.3 *(0.5)*	ns
ESS	7.7 *(1.4)*	8.0 *(2.7)*	6.1 *(1.4)*	8.9 *(1.6)*	7.7 *(1.6)*	9.7 *(1.6)*	7.4 *(1.4)*	11.3 *(1.8)*	10.5 *(1.8)*	9.7 *(0.8)*	ns
BDI	10.0 *(1.9)*	11.4 *(2.5)*	11.5 *(1.8)*	10.6 *(1.6)*	10.9 *(0.9)*	11.2 *(1.6)*	10.9 *(2.3)*	13.1 *(2.3)*	11.7 *(2.8)*	11.7 *(1.1)*	ns
BMI (kg)	27.1 *(1.5)*	25.2 *(2.3)*	25.1 *(2.0)*	24.6 *(0.8)*	25.4 *(0.8)*	23.5 *(1.2)*	25.3 *(2.2)*	24.3 *(1.0)*	24.6 *(1.9)*	24.5 *(0.8)*	ns

Table S2 Linear model overview. A. overview of the β-values from the linear model for each treatment dose indicating the cumulative average over 2- and 4-weeks **A.** all composite scores, **B.** Individual well-being items affected by PBM treatment, **C.** Individual health items affected by PBM treatment. Overall, winter and summer groups are shown. Numbers in bold represent a significant change over time (p < 0.5), bold italic represent trends (p < 0.1). Abbreviations used: ESS = Epworth Sleepiness Scale.

A

CS well-being	Overall	Winter	Summer	N
Placebo	1.98 ± 0.83	-0.46 ± 1.03	3.77 ± 1.11	13 (7:6)
1	-0.15 ± 1.15	-0.11 ± 1.46	-0.44 ± 1.52	14 (7:7)
4	-0.87 ± 1.13	-0.27 ± 1.41	-1.60 ± 1.52	15 (8:7)
6.5	0.94 ± 1.15	**3.65 ± 1.41**	-2.41 ± 1.57	14 (8:6)
**CS health**				
Placebo	-0.89 ± 0.68	-0.66 ± 0.92	-1.17 ± 0.99	13 (7:6)
1	0.92 ± 0.95	1.41 ± 1.30	0.48 ± 1.35	14 (7:7)
4	0.58 ± 0.93	0.24 ± 1.26	0.98 ± 1.35	15 (8:7)
6.5	**2.83 ± 0.95**	**3.67 ± 1.26**	1.68 ± 1.40	14 (8:6)
**CS sleep quality**				
Placebo	0.12 ± 0.56	-0.13 ± 0.79	0.41 ± 0.85	13 (7:6)
1	-1.27 ± 0.78	-0.67 ± 1.12	-1.90 ± 1.16	14 (7:7)
4	0.33 ± 0.77	0.74 ± 1.08	-0.14 ± 1.16	15 (8:7)
6.5	0.59 ± 0.78	0.83 ± 1.08	0.31 ± 1.21	14 (8:6)

B

Mood	Overall	Winter	Summer	N
Placebo	0.17 ± 0.21	-0.48 ± 0.25	0.94 ± 0.27	13 (7:6)
1	-0.00 ± 0.30	0.34 ± 0.36	-0.45 ± 0.37	14 (7:7)
4	-0.25 ± 0.29	0.20 ± 0.34	**-0.79 ± 0.37**	15 (8:7)
6.5	***0.51 ± 0.30***	**1.46 ± 0.34**	-0.64 ± 0.38	14 (8:6)
**ESS**				
Placebo	-1.27 ± 0.70	-0.28 ± 0.91	-2.41 ± 0.98	13 (7:6)
1	-0.12 ± 0.97	1.43 ± 1.29	-0.23 ± 1.34	14 (7:7)
4	0.63 ± 0.96	0.28 ± 1.24	1.06 ± 1.34	15 (8:7)
6.5	-1.09 ± 0.97	**-2.78 ± 1.25**	1.00 ± 1.39	14 (8:6)

**Subjective Performance**					
	Placebo	0.61 ± 0.37	0.43 ± 0.48	0.83 ± 0.52	13 (7:6)
	1	0.09 ± 0.51	0.21 ± 0.68	-0.05 ± 0.71	14 (7:7)
	4	-0.11 ± 0.51	-0.68 ± 0.66	0.52 ± 0.71	15 (8:7)
	6.5	-0.36 ± 0.51	0.32 ± 0.66	***-1.25 ± 0.73***	14 (8:6)

C

**IFN- γ**		**Overall**	**Winter**	**Summer**	**N**
	Placebo	0.27 ± 0.51	0.49 ± 0.68	0.01 ± 0.73	13 (7:6)
	1	-0.32 ± 0.71	-0.86 ± 0.96	0.26 ± 0.99	14 (7:7)
	4	0.26 ± 0.69	0.49 ± 0.92	-0.00 ± 0.99	15 (8:7)
	6.5	**-1.69 ± 0.71**	**-2.84 ± 0.92**	-0.18 ± 1.03	14 (8:6)
**Cortisol 0.5 h before bedtime**					
	Placebo	3.60 ± 2.78	0.79 ± 3.81	6.87 ± 4.11	13 (7:6)
	1	-4.84 ± 3.93	-2.79 ± 5.06	-7.46 ± 5.60	14 (7:7)
	4	-2.68 ± 3.86	-1.92 ± 5.38	-3.90 ± 5.60	15 (8:7)
	6.5	**-8.05 ± 3.86**	-8.25 ± 5.21	-7.31 ± 5.81	14 (8:6)
**RHR Average night after 2 and 4 weeks**					
	Placebo	1.03 ± 1.02	1.85 ± 1.34	-0.11 ± 0.16	13 (7:6)
	1	-0.66 ± 1.38	-1.42 ± 1.90	-0.43 ± 2.08	14 (7:7)
	4	-0.79 ± 1.39	-1.68 ± 1.84	0.44 ± 2.15	15 (8:7)
	6.5	-2.35 ± 1.41	**-4.60 ± 1.90**	0.45 ± 2.15	14 (8:6)

Table S3 Linear model of not significant effects overview. overview of the β-values of the linear model for the overall effect of PBM treatment as well as for the winter and summer group separately of the long term-effect analysis.

	Dose	Winter	Summer	N
**KSS**				
Placebo	-1.15 ± 0.59	-0.86 ± 0.79	-1.50 ± 0.86	13 (7:6)
1	0.29 ± 0.82	1.14 ± 1.13	-0.50 ± 1.18	14 (7:7)
4	0.85 ± 0.80	0.67 ± 1.09	1.07 ± 1.17	15 (8:7)
6.5	-0.13 ± 0.82	-0.70 ± 1.09	0.17 ± 1.22	14 (8:6)
**Need for recovery**				
Placebo	-13.28 ± 6.78	-7.79 ± 9.30	-19.69 ± 10.07	13 (7:6)
1	2.89 ± 9.40	1.95 ± 13.18	4.76 ± 13.72	14 (7:7)
4	-3.38 ± 9.25	-10.96 ± 12.76	5.41 ± 13.71	15 (8:7)
6.5	-3.92 ± 9.40	-16.64 ± 12.76	12.12 ± 14.24	14 (8:6)
**TNF-α**				
Placebo	0.02 ± 0.05	0.00 ± 0.06	0.04 ± 0.07	13 (7:6)
1	0.01 ± 0.06	-0.07 ± 0.09	0.08 ± 0.09	14 (7:7)
4	-0.06 ± 0.06	-0.02 ± 0.08	-0.11 ± 0.09	15 (8:7)
6.5	-0.08 ± 0.06	-0.03 ± 0.08	-0.13 ± 0.09	14 (8:6)
**Cortisol 3.5 h before bedtime**				
Placebo	3.09 ± 1.94	2.94 ± 2.63	3.32 ± 3.11	13 (7:6)
1	-2.87 ± 2.64	-2.38 ± 3.72	-3.42 ± 4.08	14 (7:7)
4	-1.50 ± 2.69	-2.62 ± 3.72	-0.23 ± 4.22	15 (8:7)
6.5	-2.71 ± 2.64	-2.27 ± 3.60	-3.31 ± 4.21	14 (8:6)
**aMTs6**				
Placebo	-669.3 ± 1886.7	-1028.3 ± 2736.3	-310.20 ± 2736.3	12 (6:6)
1	-607.5 ±2616.4	332.1 ± 3729.0	-1643.8 ± 3869.8	13 (7:6)
4	-253.2 ± 2616.4	-370 ± 3729.0	-56.85 ± 3869.8	14 (8:6)
6.5	-1549.5 ± 2668.2	821.6 ± 3729.0	-4725.5 ± 4058.6	12 (7:5)

Table S4 Linear model short term-effects overview. overview of the β-values of the linear model for the overall effect of PBM treatment as well as for the winter and summer group separately of the short term-effect analysis.

	Dose	Winter	Summer	N
**KSS**				
Placebo	-0.31 ± 0.58	-0.93 ± 0.80	0.42 ± 0.87	12 (6:6)
1	-0.12 ± 0.81	1.00 ± 1.14	-1.32 ± 1.18	14 (7:7)
4	-0.19 ± 0.79	0.74 ± 1.10	-1.27 ± 1.18	15 (8:7)
6.5	-0.65 ± 0.81	-0.38 ± 1.10	-0.92 ± 1.23	14 (8:6)
**Subjective performance**				
Placebo	0.08 ± 0.41	0.17 ± 0.59	0.00 ± 0.59	13 (7:6)
1	0.09 ± 0.56	0.33 ± 0.80	-0.14 ± 0.80	14 (7:7)
4	0.68 ± 0.55	0.27 ± 0.78	1.14 ± 0.80	15 (8:7)
6.5	-0.40 ± 0.56	-0.29 ± 0.78	-0.58 ± 0.83	14 (8:6)
**Cortisol 0.5 h before bedtime**				
Placebo	1.02 ± 2.76	0.45 ± 3.89	1.68 ± 4.20	13 (7:6)
1	-1.78 ± 3.84	-2.54 ± 5.50	-1.12 ± 5.73	14 (7:7)
4	0.45 ± 3.78	2.62 ± 5.33	-2.04 ± 5.73	15 (8:7)
6.5	-5.03 ± 3.84	-4.78 ± 5.73	-5.28 ± 5.73	14 (8:6)
**aMTs6**				
Placebo	-320.7 ± 1537.9	-2460.5 ± 2216.6	1819 ± 2217	12 (6:6)
1	-85.54 ± 2095.8	2246.7 ± 3020.7	-2418 ± 3021	14 (7:7)
4	-81.48 ± 2095.8	2337.5 ± 2932.2	-2594 ± 3135	14 (8:6)
6.5	-1892.6 ± 2174.9	953.2 ± 3020.7	-5021 ± 3288	12 (7:5)

Table S5 Linear model including BMI interaction overview. Secondary β-values analysis from the linear model for each treatment dose and interaction with BMI indicating the cumulative average over 2 and 4 weeks. Numbers in bold represent significant changes over time ($p < 0.5$), bold italic represent trends ($p < 0.1$)

**Need for recovery (Well-being)**		**Overall**	**Winter**	**Summer**
	Placebo	-105.4 ± 45.02	-103.70 ± 52.97	-104.63 ± 47.25
	1	108.16 ± 54.04	112.63 ± 61.63	104.06 ± 56.35
	4	75.85 ± 58.54	70.64 ± 65.83	76.45 ± 60.73
	6.5	**145.82 ± 68.81**	***141.84 ± 74.83***	**160.82 ± 71.81**
	1 x BMI	-4.12 ± 2.09	-4.13 ± 2.27	-4.13 ± 2.27
	4 x BMI	-3.07 ± 2.29	-2.97 ± 2.45	-2.97 ± 2.45
	6.5 x BMI	**-5.97 ± 2.71**	**-6.09 ± 2.87**	**-6.09 ± 2.87**
**TNF- α (Health)**				
	Placebo	0.40 ± 0.31	0.41 ± 0.35	0.41 ± 0.31
	1	-0.14 ± 0.37	-0.28 ± 0.40	-0.08 ± 0.37
	4	-0.19 ± 0.98	-0.16 ± 0.43	-0.211 ± 0.39
	6.5	**-1.09 ± 0.47**	**-1.09 ± 0.49**	**-1.16 ± 0.47**
	1 x BMI	0.00 ± 0.01	0.00 ± 0.01	0.00 ± 0.01
	4 x BMI	0.00 ± 0.01	0.00 ± 0.02	0.00 ± 0.02
	6.5 x BMI	**0.04 ± 0.02**	**0.04 ± 0.02**	**0.04 ± 0.02**

Table S6 Overview of number of reported complaints throughout the study.

	Headaches	Eye-strain	Dizziness	Tiredness	Dry skin
Placebo	1	0	1	2	0
1 PBM	1	1	0	1	1
4 PBM	0	1	0	0	0
6.5 PBM	1	1	0	0	0

Figure S1 Raw data well-being. Overview of individual items of the well-being composite score, as well as the depression scores. Left and right panels show the winter and summer group, respectively. The black dotted line represents the placebo group (n = 7:6 for winter and summer), the green line the 1 PBM group (n = 7:7 for winter and summer), the pink line the 4 PBM group (n = 8:7 for winter and summer), and the purple line the 6.5 PBM group (n = 8:6 for winter and summer). Figures show the raw data from where the deltas between week 2 and baseline and week 4 and baseline were calculated.

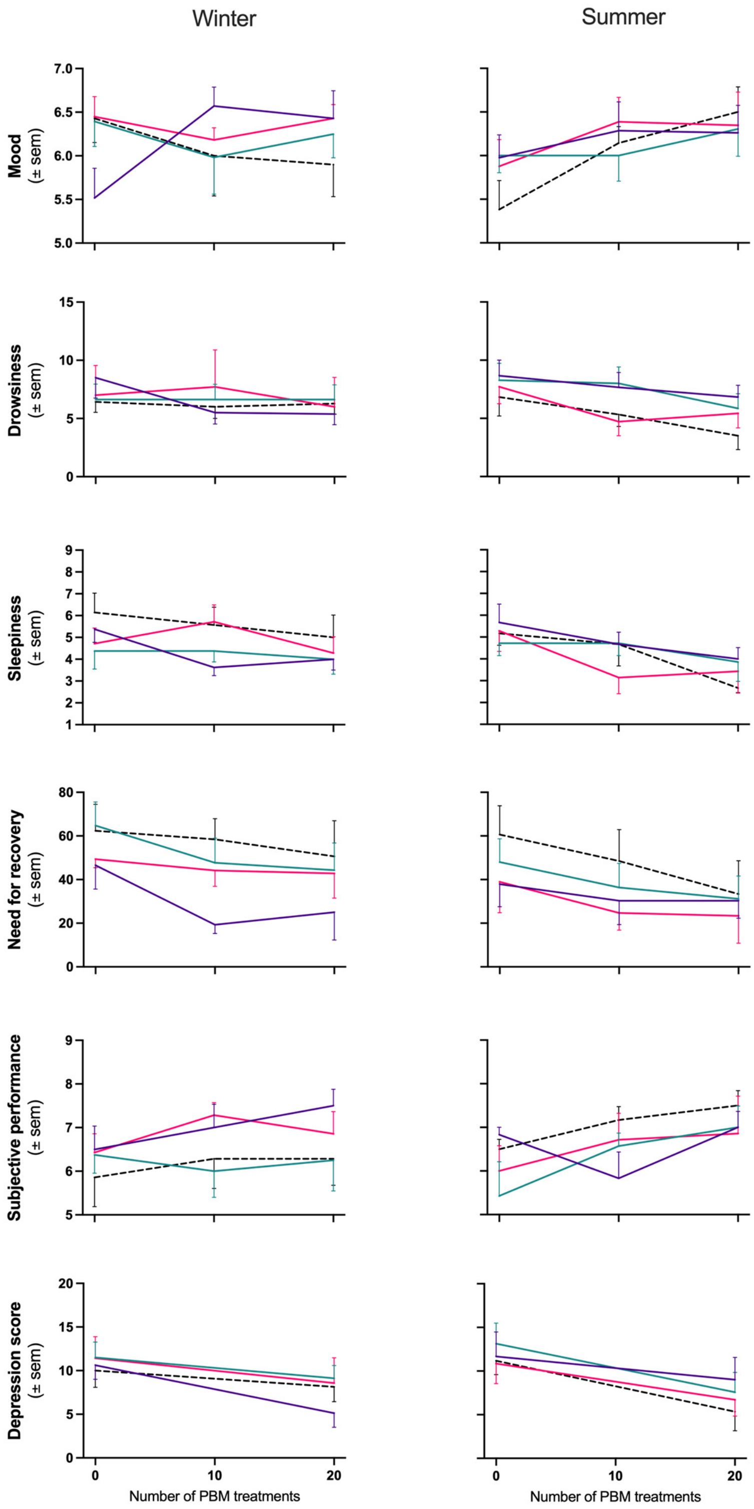

Figure S2 Raw data health. Overview of individual items of the health composite score. Left and right panels show the winter and summer group, respectively. The black dotted line represents the placebo group (n = 7:6 for winter and summer), the green line the 1 PBM group (n = 7:7 for winter and summer), the pink line the 4 PBM group (n = 8:7 for winter and summer), and the purple line the 6.5 PBM group (n = 8:6 for winter and summer). Figures show the raw data from where the deltas between week 2 and baseline and week 4 and baseline were calculated

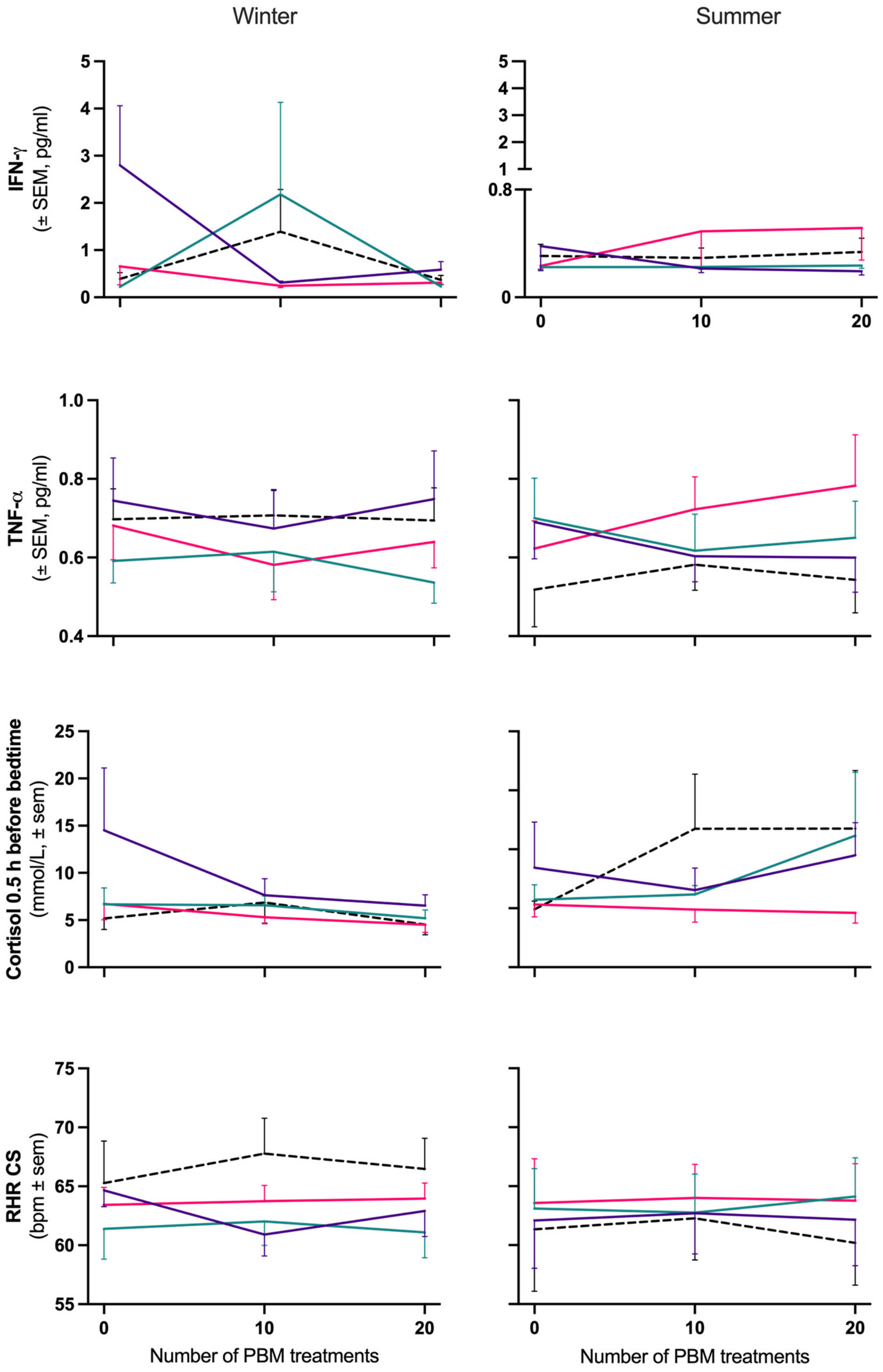

Figure S3 Raw data sleep. Overview of individual items of the sleep quality composite score. Left and right panels show the winter and summer group, respectively. The black dotted line represents the placebo group (n = 7:6 for winter and summer), the green line the 1 PBM group (n = 7:7 for winter and summer), the pink line the 4 PBM group (n = 8:7 for winter and summer), and the purple line the 6.5 PBM group (n = 8:6 for winter and summer). Figures show the raw data from where the deltas between week 2 and baseline and week 4 and baseline were calculated.

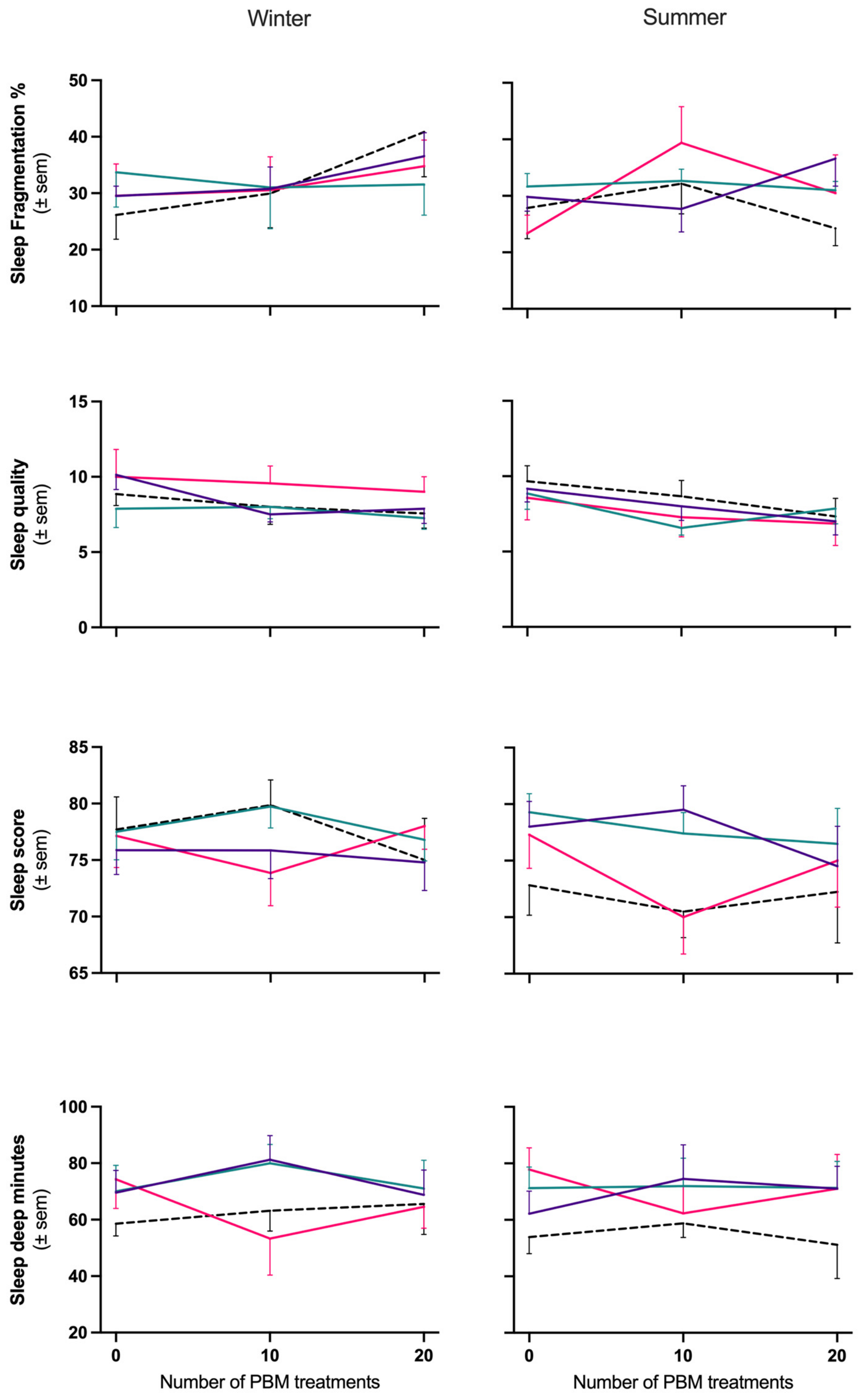

Figure S4 Ambient light and Vitamin D. A. Ambient light exposure: average and range of ambient light intensity (log lux) during the PBM treatments measured by the lux sensor at the back of the PBM module, for all PBM conditions. **B.** The average changes in 25-OH-Vitamin D3 over 4 weeks in winter and summer for all PBM conditions. Significance codes: *** p < 0.001, ns: not significant. Sample size per condition is shown.

A

B

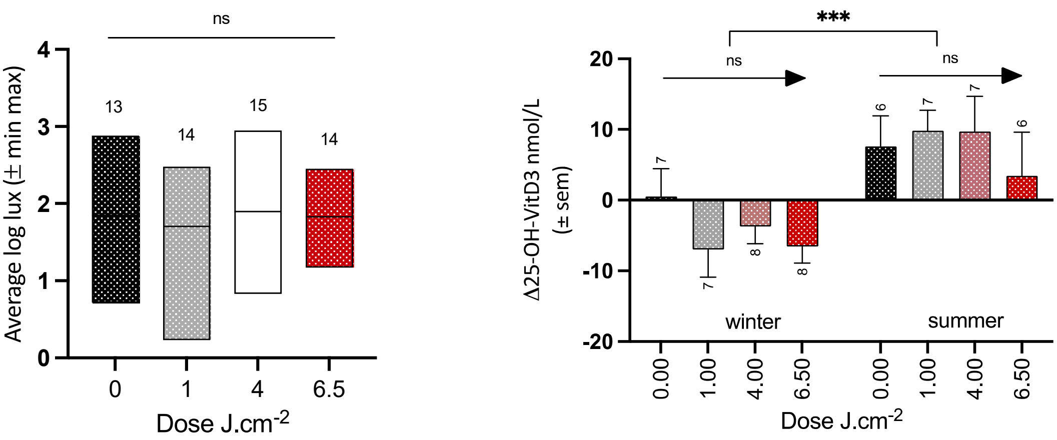

Figure S5 Depression scores. Average change in BDI values for all conditions for winter and summer separately. The tendency for an interaction between season and 6.5 PBM condition is depicted by #. Significance codes: # < 0.1, ns: not significant. Sample size per condition is shown.

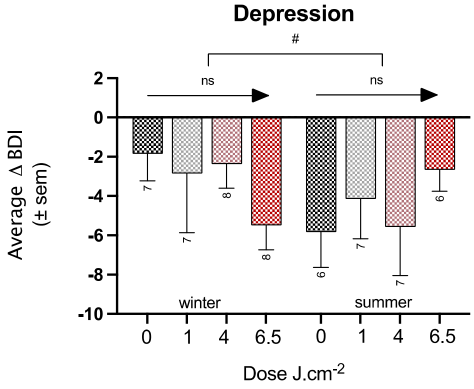

Figure S6 Skin temperature. Average z-transformed skin temperature for **A.** 1 J. cm^{-2} PBM dose, **B.** 4 J. cm^{-2} PBM dose and **C.** 6.5 J. cm^{-2} PBM dose. F: finger, A: ankle, H: head and C: claviculae. Significance codes: * < 0.05.

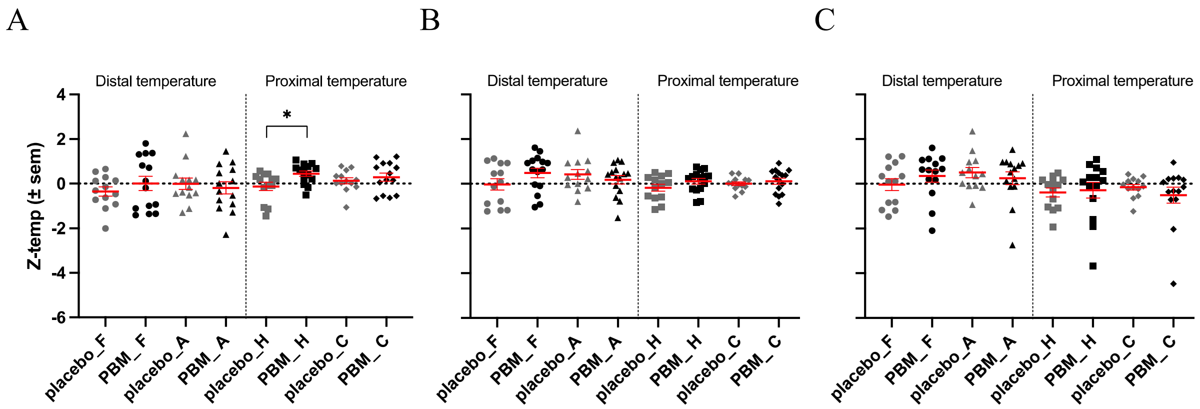

Supplement: Supplementary file 1 [file biology-12-00060-s001.zip › biology-2050450-supplementary.pdf]
